# Aggregation Behavior of Medium Chain Fatty Acids Studied by Coarse-Grained Molecular Dynamics Simulation

**DOI:** 10.1208/s12249-018-1289-4

**Published:** 2019-01-09

**Authors:** Md Shakhawath Hossain, Staffan Berg, Christel A. S. Bergström, Per Larsson

**Affiliations:** Dept of Pharmacy and the Swedish Drug Delivery Forum (SDDF), Uppsala University, Uppsala Biomedical Center, P.O. Box 580, SE-751 23 Uppsala, Sweden

**Keywords:** medium chain fatty acid, aggregation, critical micelle concentration, coarse-grained molecular dynamics, fatty acid aggregation behavior, experiment simulation comparison

## Abstract

Medium chain fatty acids (MCFA) are digestion products of lipid-rich food and lipid-based formulations, and they are used as transient permeability enhancers in formulation of poorly permeable compounds. These molecules may promote drug absorption by several different processes, including solubilization, increased membrane fluidity, and increased paracellular transport through opening of the tight junctions. Therefore, understanding the aggregation behavior of MCFAs is important. A number of studies have measured the critical micelle concentration (CMC) of MCFAs experimentally. However, CMC is highly dependent on system conditions like pH, temperature, and the ionic strength of the buffer used in different experimental techniques. In this study, we investigated the aggregation behavior of four different MCFAs using the coarse-grained molecular dynamics (CG-MD) simulations with the purpose to explore if CG-MD can be used to study MCFA interactions occurring in water. The ratio of deprotonated and non-charged MCFA molecules were manipulated to assess aggregation behavior under different pH conditions and within the box sizes of 22 × 22 × 44 nm^3^ and 44 nm^3^ for 1 μs. CMCs were calculated by performing CG-MD simulations with an increasing number of MCFAs. The resulting aggregate size distribution and number of free MCFA molecules were used to determine the CMC. The CMCs from simulations for C_8_, C_10_, and C_12_ were 1.8–3.5-fold lower than the respective CMCs determined experimentally by the Wilhelmy method. However, the variation of MCFA aggregate sizes and morphologies at different pH conditions is consistent with previous experimental observation. Overall, this study suggests that CG-MD is suitable for studying colloidal systems including various MCFAs.

**Electronic supplementary material:**

The online version of this article (10.1208/s12249-018-1289-4) contains supplementary material, which is available to authorized users.

## INTRODUCTION

One of the most successful approaches in promoting the absorption of poorly soluble drugs through the intestinal epithelium has been the use of lipid-based enhancers primarily based on medium chain fatty acids (MCFA) [1]. MCFAs can increase the epithelial flux through transient transcellular perturbation as well as paracellular pathways [1–5]. The effect of MCFAs on the intestinal epithelium is transient and rapidly reversible [6, 7]. Therefore, MCFAs are ideal components for transient permeability enhancers for peptides, proteins, macromolecules, and oral formulations [1, 6]. Transient permeability enhancers have become an essential component of a number of oral formulations including oral peptide and oligonucleotide dosage forms currently undergoing clinical trials [8–12]. MCFA and MCFA derivatives are used in solid dosage forms, and is commonly known as a gastrointestinal permeation enhancement technology (GIPET) [11]. It has been tested with various doses with poorly absorbed drugs in 16 Phase I studies for which oral bioavailability of 5–13% was observed when GIPET dosage forms were used in comparison to the bioavailability of less than 1% that is typically achieved [11]. Furthermore, MCFAs are common in food products and may be generated from glycerides during lipid digestion. No significant toxicity effect has been attributed to the use of these lipid-based transient permeability enhancers, even with repeated dosing [8, 11, 13].

In aqueous solution, fatty acid molecules are able to self-assemble and micelles of various sizes and structures can form depending on fatty acid concentration, which may affect the efficiency of MCFAs as transient permeability enhancers, because free fatty acid monomers present near the intestinal absorption site can directly impact the extent of transient permeability [1]. Therefore, it is important to estimate the number of fatty acid monomers in both free and aggregate forms at a given concentration in order to identify the threshold concentration required to enhance absorption. Typically, the critical micelle concentration (CMC) is considered a good criterion for threshold concentration [1, 14]. However, the aggregation behavior and CMC value of different MCFAs are highly dependent on various system properties such as pH, temperature, and ionic strength [1, 14]. A large number of studies have investigated aggregation behavior and determined the CMC values of MCFAs using different experimental techniques such as surface tension measurement [14], the dye micellization method [15], the relative viscosity-based method [16], high resolution ultrasonic spectroscopy [6], ion activity measurement, and conductimetry techniques [17]. One key observation regarding the experimentally measured values is that CMC values also depend on the methods of measurement: the difference between CMC values measured using different techniques at similar or specific system conditions is relatively large.

Molecular dynamics (MD) simulations have shown significant promise for investigating the aggregation behavior of different surfactant molecules [18–20]. However, the atomistic all atom (AA) model [21, 22] in which each atom is represented individually has limited capabilities for measuring equilibrium properties like CMC and aggregate size distribution, since these methods are generally confined to nanosecond time scales and nanometer length scales [20]. Sammalkorpi et al. [23] estimated the CMC of sodium hexyl sulfate (S6S) using an atomistic MD simulations with a united atom model. In this system, 200 ns was sufficient for the surfactant model to reach an equilibrium state. For MCFAs and surfactants like sodium dodecyl sulfate (SDS), such a short time scale is insufficient for fully equilibrating the system [23]. One approach for extending the box size and time scale of experiments is using coarse-grained (CG) MD simulations [24]. In the CG-MD approach, a number of atoms are represented by a single CG bead, which significantly reduces the number of interaction sites in a simulation [24–26]. Therefore, these simulations can be performed for relatively longer times and greater length scales, but at the cost of sacrificing the atomic resolution allowed by the AA model [27]. CMC values determined using CG-MD for different surfactants like dodecylphosphocholine (DPC) [28], and S6S [29], sodium nonyl sulfate [29], and SDS [29, 30] were found to have reasonable agreement with experimental values. Results using the dissipative particle dynamics (DPD) also agreed quantitatively with experimental CMC and aggregation number for several surfactants such as octaethylene glycol monooctyl ether, dodecyldimethylamineoxide (DDAO), and *N*-decanoyl-*N*-methyl-D-glucamine [31, 32]. However, an important limitation of DPD is the limited availability of verified model parameters. In CG-MD, the parameterization issue is greatly reduced by, *e.g.*, the Martini model developed by Marrink et al. [25, 26]. In the Martini model framework, a specific molecule can be constructed relatively easily by using the different particle types and interactions described in the model [25]. The model was used to determine the CMC value of H_1_T_4_ and tetra(ethylene) glycol monoalkyl ether (C_8_E_4_) and was found to overpredict the experimental values by a factor of 5 and 4 respectively [20]. However, the model was able to capture the aggregate size distribution and average aggregate size for H_1_T_4_ and C_8_E_4_ [20], as well as complex morphological changes of the oleic acid aggregates based on the protonation state of the oleic acid head groups [33] and complex phase behavior of oleic acid including the pKa shift from 4.8 in water to 6.5 in small micelle and 6.6 in bilayer [34].

The aim of this study was to investigate CMC and aggregation behavior of four MCFAs (C_8_, C_10_, C_12_, and C_14_) as a function of protonation state of those respective MCFAs and the ionic strength of the buffer using CG-MD simulations with the Martini model. To verify how closely the CG-MD reproduced the experimental CMC values, we also measured the CMCs of C_8_, C_10_, and C_12_ experimentally at different representative system conditions by mimicking our simulation conditions as closely as possible. The aggregation behavior obtained from the simulation was also compared with different experimental observations available in the literature.

## METHODS

### CG Model and Simulation

The simulations of MCFA aggregates were run using the CG Martini v2.0 model [26]. In this model, typically two to four heavy atoms are represented by one CG bead. To model C_14_ (which has 14 carbon chain with a carboxyl headgroup), we used the available MCFA topology from the cgmartini.nl website, with three C1 beads used to represent the carbon chain and one bead (P4 or Qa) was used to represent the carboxylic head group, either as neutral or with a negative charge. The model of C_10_ was obtained from C_14_ by excluding one CG bead from the carbon chain, since one CG bead typically represented four heavy atoms. Therefore, the CG model of C_10_ included three beads with P4/Qa for the headgroup and two beads for representing the carbon chain. The four-to-one mapping of heavy atoms into beads is both a strength and a weakness of the Martini model. One consequence is that it is not possible to separate, *e.g.*, fatty acids with similarly long carbon chains from each other. In this context, we, however, sought to investigate whether it was possible to circumvent and obtain relevant CMC values. To model C_12_ which has 12 carbon chains with a carboxyl headgroup, we used four CG beads similar to C_14_. However, to represent the reduced chain length of C_12_ compared to C_14_, the effective inter-particle distance of the beads was reduced from 0.47 to 0.37 nm, and a similar change was made going from C_10_ to C_8_. No other changes were made to the force field. The ratio of non-charged and deprotonated molecules was varied to simulate the effect of pH on the MCFA aggregation (Table I).

**Table I:** CMC Values (in mM) of the MCFAs for Different Systems. Here, (H)^−1^ and (H)^0^ Represent the MCFA Molecules with Negatively Charged and Non-charged Headgroups Respectively. A Single Asterisk Indicates a CMC Value Obtained from Simulation Performed at 50°C

	(H)^−1^	(H)^−1^ 140 mM NaCl	(H)^−1^:(H)^0^ (1:1) 140 mM NaCl	(H)^0^ 140 mM NaCl	Experimental
C_8_	38.69	31.68	13.88	9.26	75 (at pH = 12) (this work) 42.1 (at pH = 7) (this work) 75–300 [6, 35]
C_10_	18.68	14.89	8.27	5.08	45.7 (at pH = 12) (this work) 15.5 (at pH = 7) (this work) 26–100 [1, 6, 14, 16]
C_12_	1.64	1.33 1.71^(^*^)^	1.09	1.15	5.97 (at pH = 12) (this work) 1.58–30 [6, 14, 16]
C_14_	0.99	0.86	0.83	0.77	0.23–4 [14, 17]

MD simulations were performed in Gromacs 2016.4 [36] with a 30-fs time step. Isotropic pressure coupling with a reference pressure of 1 bar (1 bar = 100 kPa) was maintained with the Berendsen coupling method. Temperature was set at 37°C for most of the simulation. One set of simulation for C_12_ was performed at 50°C for the purpose of mimicking the experimental condition. Non-bonded interactions were set according to the recommendations for the Martini force fields and Gromacs 2016.4 version. For the simulations of C_8_ and C_10_ aggregates, the length, width, and height of the box was 22 nm, 22 nm, and 44 nm respectively. To represent the concentration of 1 mM of MCFA in the simulation box, 13 fatty acid molecules were needed. Approximately 192,000 water beads were used to fill up the box with water. When MCFA were used in the deprotonated state, an equal amount of positively charged sodium ions were also included to make the system neutral. To represent an ionic strength of 140 mM NaCl, 1820 Na^+^ and Cl^−^ ions were added in the simulation box. For a number of simulations with C_12_ and C_14_, a larger box of 44 nm^3^ was used, because the aggregation occurs at much lower concentrations and the larger box size was required for reliable calculation of the number of free and aggregated molecules. To determine the CMC of a particular system, a number of simulations with increasing number of MCFA molecules were performed initiated from random dispersion of molecules in the box. Prior to the production run, energy minimization was performed for 10,000 steps using the steepest descent algorithm for each system followed by four short equilibration runs (50,000 steps) with time steps of 1, 2, 5, and 20 fs, respectively.

### MCFA Aggregates Determination

**Fig. 1:**
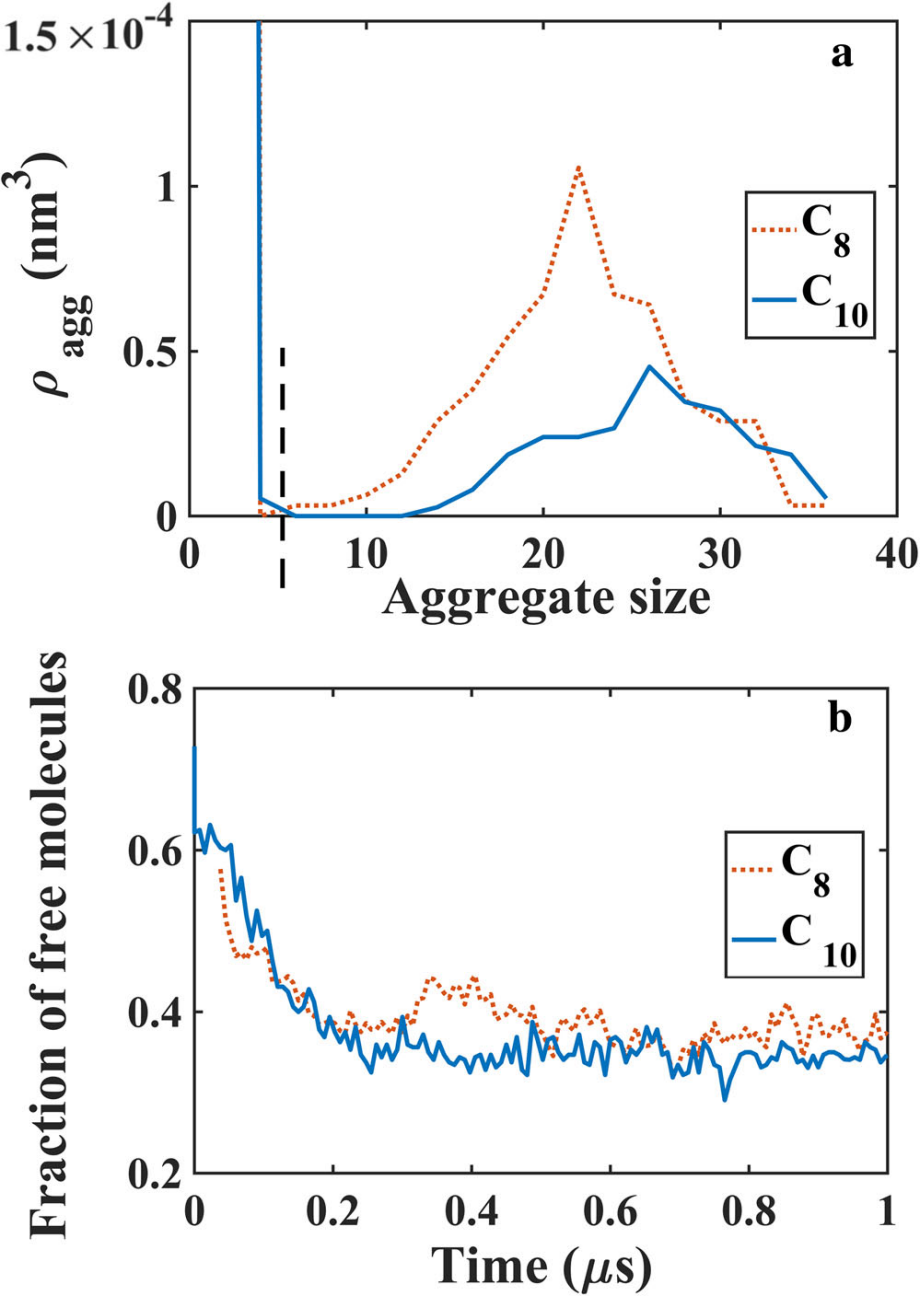
**a** Normalized aggregate size distribution and **b** fraction of free molecules for C_8_ (50 mM) and C_10_ (25 mM). The dashed vertical line in **a** indicates the cutoff size between free and aggregated molecules. All the C_8_ and C_10_ molecules were in the deprotonated state and no additional ionic concentrations were used

To determine the MCFA aggregates, two MCFA molecules were considered to be in the same aggregate if their constituent beads were within a specific distance from each other. This cutoff distance was determined to be 0.6 nm for the Martini model [20]. The cutoff size for the free MCFA molecules was selected from the aggregate size distribution and considered to lie between the first two peaks of the distribution [20]. Typical aggregate size distribution for C_8_ and C_10_ is shown in Fig. 1a, indicating a cutoff aggregate size of 5 to be reasonable. The aggregate size distribution for C_12_ and C_14_ is shown in Supplementary Fig. S1. It is possible that the boxes did not include enough molecules for obtaining a complete aggregate size distribution, but Supplementary Fig. S1 shows a trend similar to that of Fig. 1a, so cutoff size 5 was used for C_12_ and C_14_ as well.

### CMC Definition and Determination

Different definitions of CMC are currently in use, including the concentration at which micelles start to appear, or the concentration at which half of the molecules are in the aggregate form and half are free [20]. This study is based on the second definition. The number of free MCFA molecules were calculated over the simulation trajectory as shown in Fig. 1b. The fraction of free molecules (averaged over 20 snapshots within 0.9 to 1 μs of the simulation time) was used to determine CMC.

### CMC Surface Tension Measurements

The surface tension measurement utilizing the Wilhelmy plate is the most common CMC measurement technique [14, 15, 37]. This method is also known as the Wilhelmy method. In this method, the surface tension of aqueous solution with surfactants is measured by using a platinum plate placed inside the solution. The surface tension value decreases with increasing surfactant concentration in the solution and becomes constant above the CMC [15].

In our experiment, the surface tension of phosphate buffer containing MCFA was measured on a Sigma 70 instrument to determine CMC. Measurement temperatures were 37 °C for C_8_, C_10_, and 50°C for C_12_ in order to stay above the Krafft point. Buffer solutions were designed to have ionic strengths of 140 mM. Two different pH values of 7 and 12 were used when measuring the surface tension to mimic the desired system conditions. All measurements were performed in triplicate.

## RESULTS

**Fig. 2:**
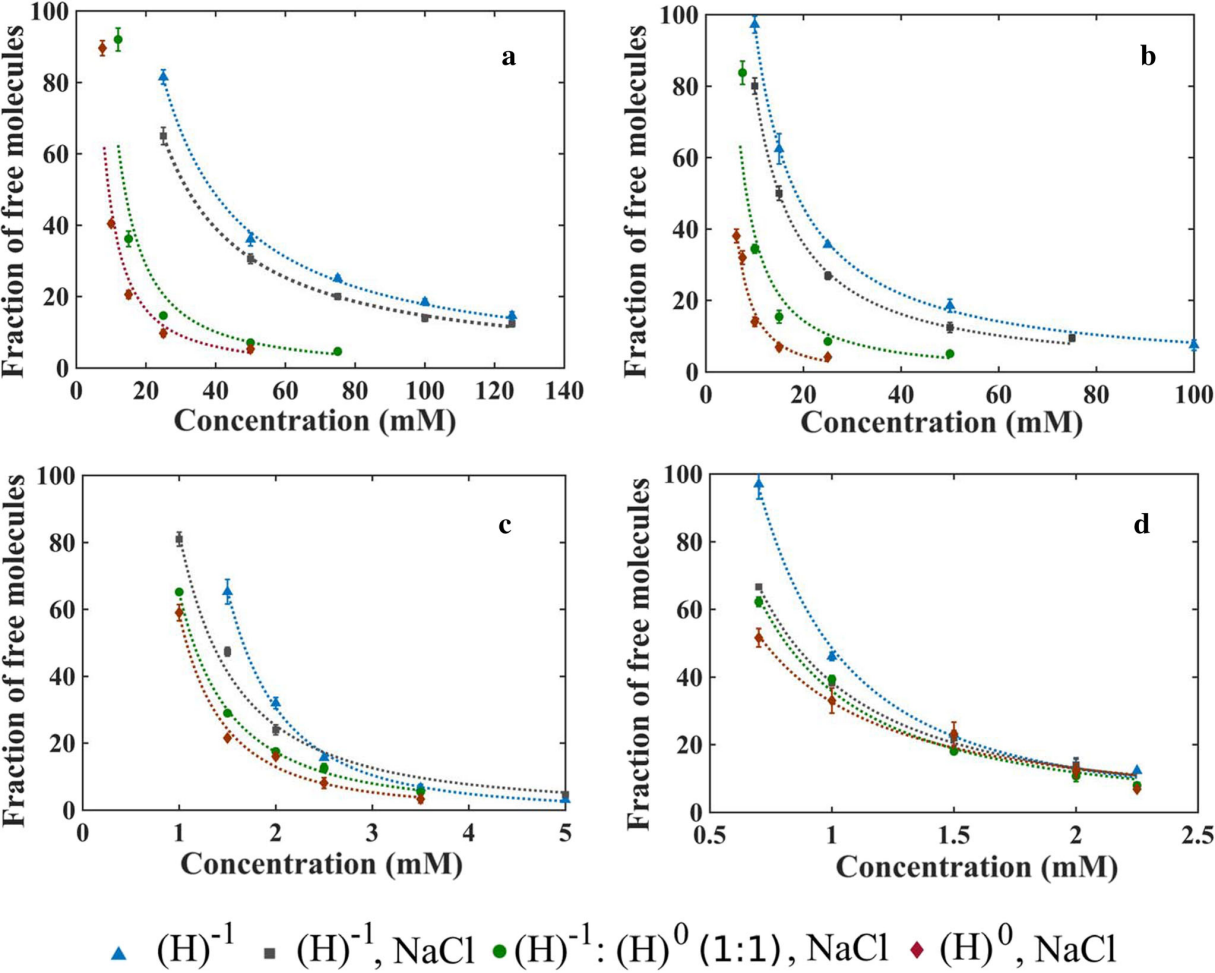
Fraction of free molecules at different concentration levels for **a** C_8_, **b** C_10_, **c** C_12_, and **d** C_14_. Here, (H)^−1^ and (H)^0^ represent the MCFA molecules with negatively charged and non-charged headgroups respectively. The dotted line shows the fitting with the power-law equation

To determine CMC using CG-MD, we first calculated the fraction of free molecules at different concentrations for each MCFA in specific system conditions (Fig. 2). The free fraction of molecules was generated based on 20 snapshots from the simulations, and the average value obtained from these snapshots was used for CMC calculation. Each set of values was fitted with a nonlinear power-law equation which was then used to determine the concentration at which 50% of the molecules are free in the simulation, and this concentration value was taken as the CMC value (see Methods; Table I). The *R*-squared values for fitting equations were 0.941–0.995.

For each MCFA, the largest CMC value was obtained when all molecules were in the deprotonated state and no other ions were present in the system (Table I and Fig. 2). Including 140 mM NaCl with the deprotonated MCFA molecules decreases the CMC values. The lowest CMC value was observed for all four MCFAs when all the molecules were at the non-charged state in the presence of 140 mM NaCl (note that using all the molecules in the deprotonated and non-charged state mimics high and low pH conditions respectively). CMC values for the systems with 1:1 ratios of deprotonated and non-charged molecules were higher than CMC values for the system with 100% non-charged molecules, but lower than the system with 100% deprotonated molecules, in line with what has been experimentally observed [18]. The CMC values of the different MCFAs decreased with increasing carbon chain length. This relationship was consistent for all four different systems. The CMC values obtained for C_8_ and C_12_ using the reduced inter-particle distance showed good consistency with respect to the values obtained for C_10_ and C_14_ which used the actual bead inter-particle distance of the Martini model.

**Fig. 3:**
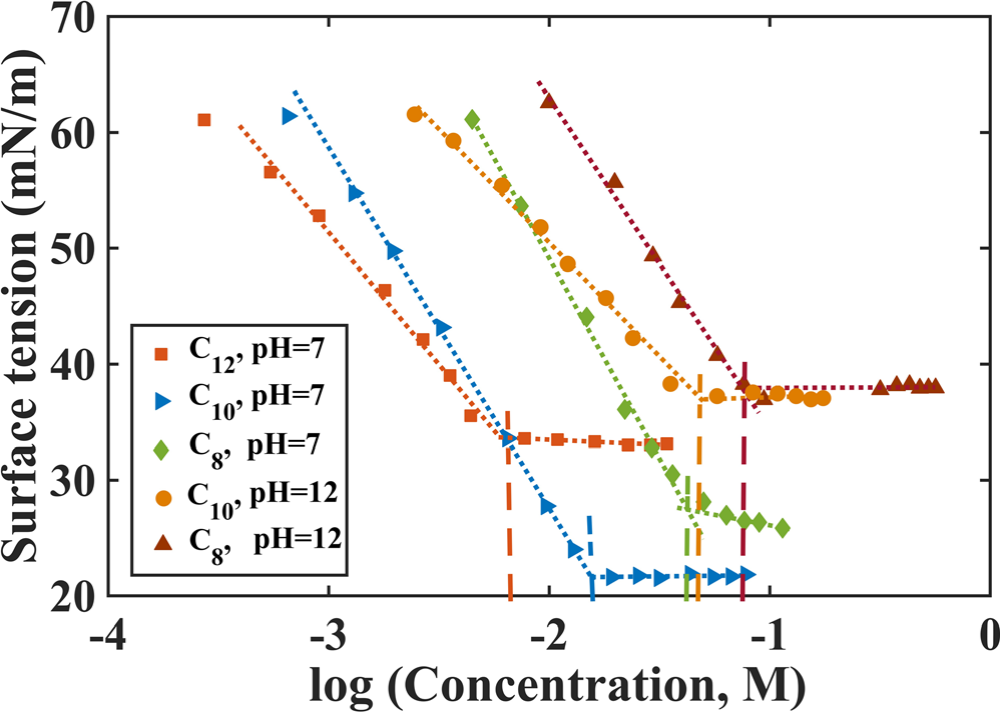
Surface tension (mN/m) *vs* log MCFA concentration (M) for C_8_, C_10_, and C_12_. The dotted line shows the fitting of the linear parts of the data set using linear regression by least squares. The corresponding concentration of the intersection of the two lines for each MCFA as indicated by the dashed lines was considered to be the CMC

We also measured the CMCs of C_8_, C_10_, and C_12_ experimentally to validate the exact model configuration used in this study, with particular interest in capturing the effect of decreased bead interaction distance used in the C_8_ and C_12_ model. These experimentally determined CMC values are in Table I. To determine these CMCs using the Wilhelmy method, the surface tensions as a function of concentration were measured (Fig. 3). The linear parts of the data sets for each MCFA were fitted using linear regression by least square. The corresponding concentration of the intersection point between the two fitted lines was considered to be the CMC.

The experiments performed at pH = 12 with 140 mM NaCl corresponds to the simulated system with 100% deprotonated MCFA molecules with ionic strength. The pKa values for C_8_ and C_10_ are slightly above 7 [35]. Therefore, the experiments performed at pH = 7 and 140 mM NaCl corresponds to the simulated system with a 1:1 ratio of deprotonated and non-charged fatty acid molecules and NaCl. To compare experimentally determined CMC for C_12_, we performed a new set of simulations at 50°C with 100% deprotonated molecules and 140 mM NaCl. Based on the fraction of free molecules obtained in the new simulations (Supplementary Fig. S2), the CMC value was calculated to be 1.71 mM (Table I).

**Fig. 4:**
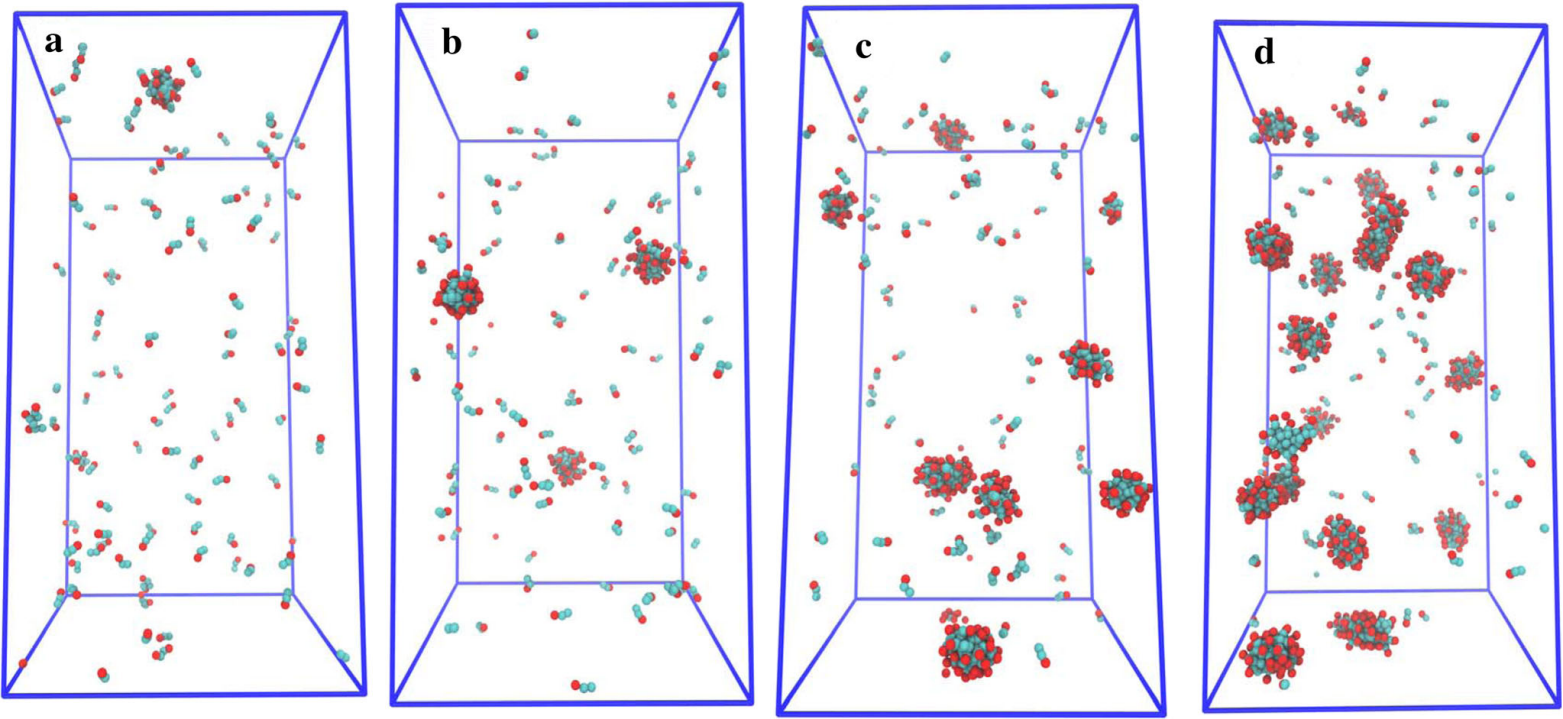
Snapshots of the simulations using **a** 10, **b** 15, **c** 25, and **d** 50 mM C_10_ in the system with 100% deprotonated molecules and 140 mM NaCl. The red and cyan color represent the headgroup and carbon chain beads, respectively

Figure 4 shows snapshots of simulations of C_10_ at different concentrations for the system with 100% deprotonated molecules and 140 mM NaCl. For this system, calculated CMC was 14.89 mM (Table I). The number of free C_10_ molecules is clearly very high in the simulation snapshot (Fig. 4a) of 10 mM C_10_. As the concentration of C_10_ increases, the decreasing number of free molecules and increasing number of C_10_ aggregates are clearly indicated by the simulation snapshots.

**Fig. 5:**
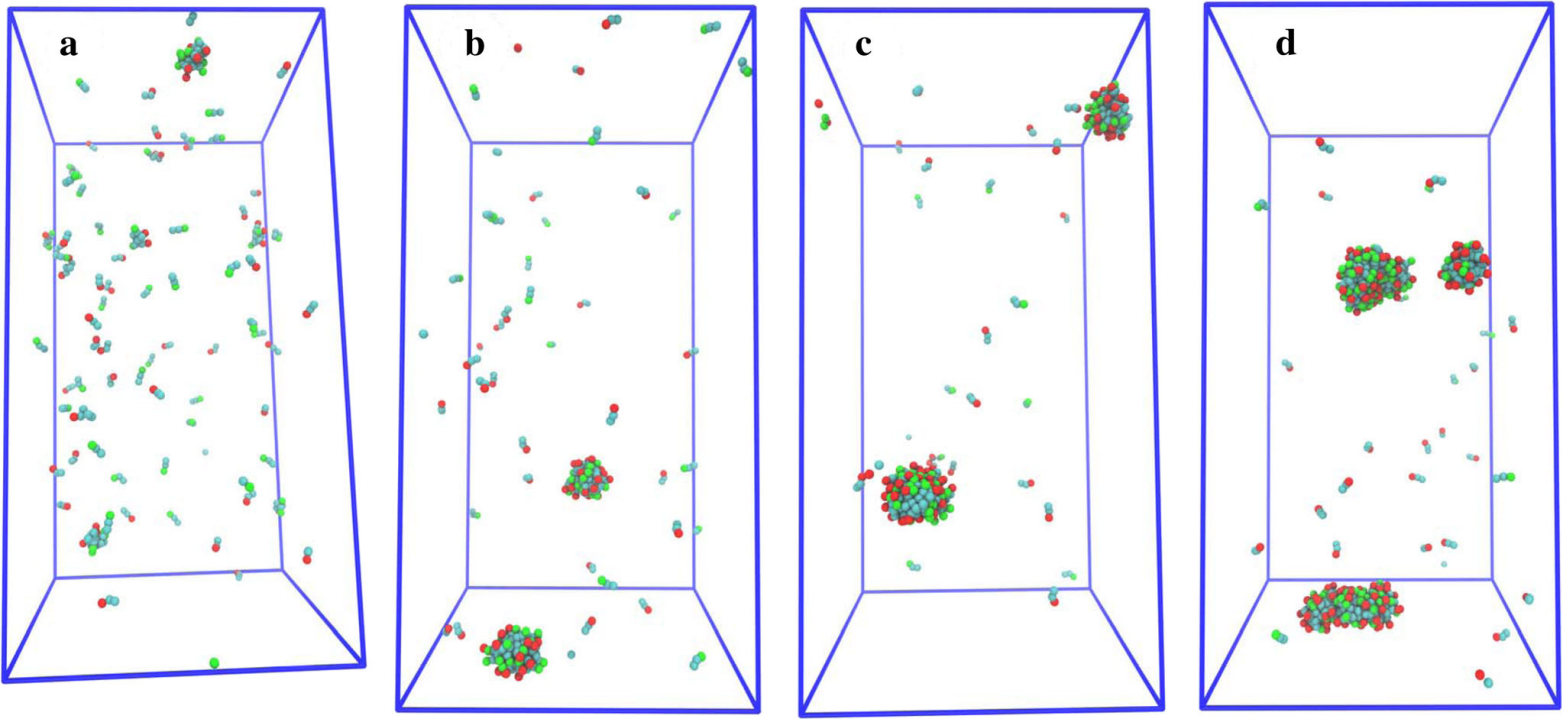
Snapshots of the simulations using **a** 7.5, **b** 10, **c** 15, and **d** 25 mM C_10_ in the system with a 1:1 ratio of deprotonated and non-charged molecules and 140 mM NaCl. The red and green color represent the headgroup beads as negatively charged and neutral, respectively. The cyan color represents the carbon chain beads

Simulation snapshots of C_10_ at different concentration levels for the system with a 1:1 ratio of deprotonated and non-charged molecules and 140 mM NaCl are shown in Fig. 5. The CMC of C_10_ for this system was calculated to be 8.27 mM (Table I). The snapshots demonstrate that the fraction of free molecules decreases as the concentration increases (similar to the 100% deprotonated system). However, this system has fewer aggregates and a larger aggregate size compared to the 100% deprotonated system at the same concentration level. The snapshots with the C_10_ concentration higher than CMC (Fig. 5c, d) also indicate that a majority of the free molecules are deprotonated.

## DISCUSSION

Previous studies have used the free monomer concentrations from simulations performed with high surfactant concentrations to determine CMCs, on the assumption that the monomer concentration does not vary much above the CMC [28, 31], but this approach has been shown to not be suitable for surfactants with relatively low CMC values (less than 60 mM) [29]. A theoretical model was derived from the free energy density of a system containing free monomers, micelles, and counterions to calculate CMC; however, this approach was also shown to underestimate CMC values of various surfactants [30]. CMC has also been predicted from its relationship with Gibbs free energy and temperature [20]. Using this method, CMC values for nonionic surfactants were closer to the measured values, but for zwitterionic surfactants, CMCs were again significantly underestimated [20].

In this work, we used a more direct approach based on [20], using CG-MD simulations and a free fraction of molecules of 50% as the CMC value. This method resulted in CMC values consistent with previously reported results such as the observed decrease in CMC values with increasing carbon chain length has been observed in a number of experimental measurements [6, 37, 38]. The decrease in CMC values with increasing ionic strength has also been experimentally determined for various surfactants, including C_12_ [39], SDS [39], DDAO [40], and including bile salts [41]. We observed that the protonation state of the MCFA concentrations affects CMC values. At the same ionic strength, when the system changed from 100% molecules with deprotonated carboxylic head groups to a 1:1 ratio of deprotonated to protonated carboxylic head groups, CMC values decreased 56% for C_8_, 44% for C_10_, 14% for C_12_, and 11% for C_14_. The systems with all molecules with neutral and deprotonated head groups mimic low and high pH, while the systems with 1:1 charged and non-charged head groups mimics the pH equal to the pKa of the molecule [33]. The effect of pH has previously been experimentally investigated for SDS [42] and decanoic acid/decanoate [18], where it was found that CMC increased with increasing pH, which correlates well with our simulated values in different system conditions mimicking different pH levels. MCFAs also form bilayers or vesicles in the presence of uncharged molecules above the critical concentration for vesicle formation (CVC) [43]. A number of studies have investigated the transition between vesicles and micelles at various system pH values and ratios of deprotonated and neutral molecules, and have found that MCFAs form bilayers at low and moderate pH values and micelles at high pH values [44, 45]. We noticed similar pH-dependent morphologies of the aggregates for C_8_ (bilayers at low/intermediate pH, micelles at high pH; snapshots in Supplementary Fig. S3).

In addition to the simulations described above, we also measured CMC of C_8_, C_10_, and C_12_ at two different system conditions (Table I, section 3) using the Wilhelmy method. The measured CMC values also showed decrease with increasing chain length of the MCFA molecules and decreasing pH of the system. Our simulations underestimated the experimentally measured CMCs by a factor of 1.8 to 3.5. This discrepancy was probably due to limitations of the CG force field used in this study, which is not capable of truly representing a specific pH condition of the buffer solution (water in this case). Note that the CMC is highly dependent on the buffer solution of the system. Different experimental methods used to measure the CMC also provided large variation in results mainly due to the variation of the measurement methods and the use of different buffer solutions [6, 14, 16, 45].

The key finding of this study was to demonstrate the viability of the CG-MD simulation technique in determining aggregation and CMCs of MCFAs at different system conditions. The close correlation between the simulation results and experimental data suggests that the CG-MD technique may be a suitable candidate for studying fatty acids as formulation candidates and permeability enhancers for the delivery of oral macromolecular drugs. This technique captured the small size difference among the various MCFAs by the very simple change of introducing a reduced CG bead-bead distance. This modification of the CG Martini model seems to provide results consistent with the experimental values, but this approach requires careful validation for other systems and research questions. The major advantage of the CG-MD technique is that it requires fewer computational resources than the AA-MD simulations. Therefore, relatively large systems can be simulated over longer time scales. Our simulations needed around a microsecond to reach equilibrium so that the number of molecules in the free and aggregated forms were stable. We also needed a larger than usual box size (44 nm^3^) for the simulations of C_12_ and C_14_ in order to maintain an adequate number of MCFA molecules so that aggregation could occur. We performed a few simulations by varying the box sizes but with the same ratio of MCFA molecules and box size to determine the fraction of free molecules in each case. We were able to verify that five times more molecules than the cutoff value of the aggregate size is sufficient to obtain reliable fraction of free molecules.

## CONCLUSIONS

In this study, we investigated the aggregation behavior of four MCFAs commonly used as transient permeability enhancers for oral delivery of macromolecules. Our CMC measurements from CG-MD simulations were 1.8- to 3.5-fold lower than the experimentally measured values. The aggregate size, numbers, and morphologies as a function of carbon chain length, MCFA concentration, and ratio of deprotonated and non-charged MCFA molecules was consistent with experimental data. The calculated CMC values also ranked the compounds similarly as the experimental values. However, in order to reproduce the experimental CMC values more closely, improved force field parameterization to better represent the exact pH value of the buffer system might be needed. Among the current available models, polarizable water model can be used to better represent the electrostatics of the system. Finally, this study suggests that the CG-MD Martini model is promising for evaluating MCFAs as formulation candidates and transient permeability enhancers.

## Electronic supplementary material

Supplementary Figure 1 Normalized aggregate size distribution for C_12_ (5 mM) and C_14_ (1 mM). The dashed vertical line indicates the cutoff size between free and aggregated molecules. All the C_12_ and C_14_ molecules were in the deprotonated state and no additional ionic concentrations were used. (PNG 3256 kb)

High Resolution Image (TIF 805 kb)

Supplementary Figure 2 Fraction of free molecules at different concentration level for C_12_ at 50C with 100% deprotonated molecules and 140 mM NaCl. The dotted line shows the fitting with the power-law equation. (PNG 190 kb)

High Resolution Image (TIF 5321 kb)

Supplementary Figure 3 Snapshot of the simulations of 50 mM C_8_ with 140 mM NaCl for the systems with (a) 100% deprotonated molecules, (b) 1:1 ratio of deprotonated and non-charged molecules and (c) 100% non-charged molecules. The red and green represent the headgroup beads as negatively charged and neutral, respectively. The cyan color represents the carbon chain beads. (PNG 17809 kb)

High Resolution Image (TIF 19859 kb)
