# Supplementary figures and images for: Aggregation Behavior of Medium Chain Fatty Acids Studied by Coarse-Grained Molecular Dynamics Simulation
Source: AAPS PharmSciTech. 2019 Jan 9;20(2):61. doi: 10.1208/s12249-018-1289-4 (PMC6373435; doi:10.1208/s12249-018-1289-4)

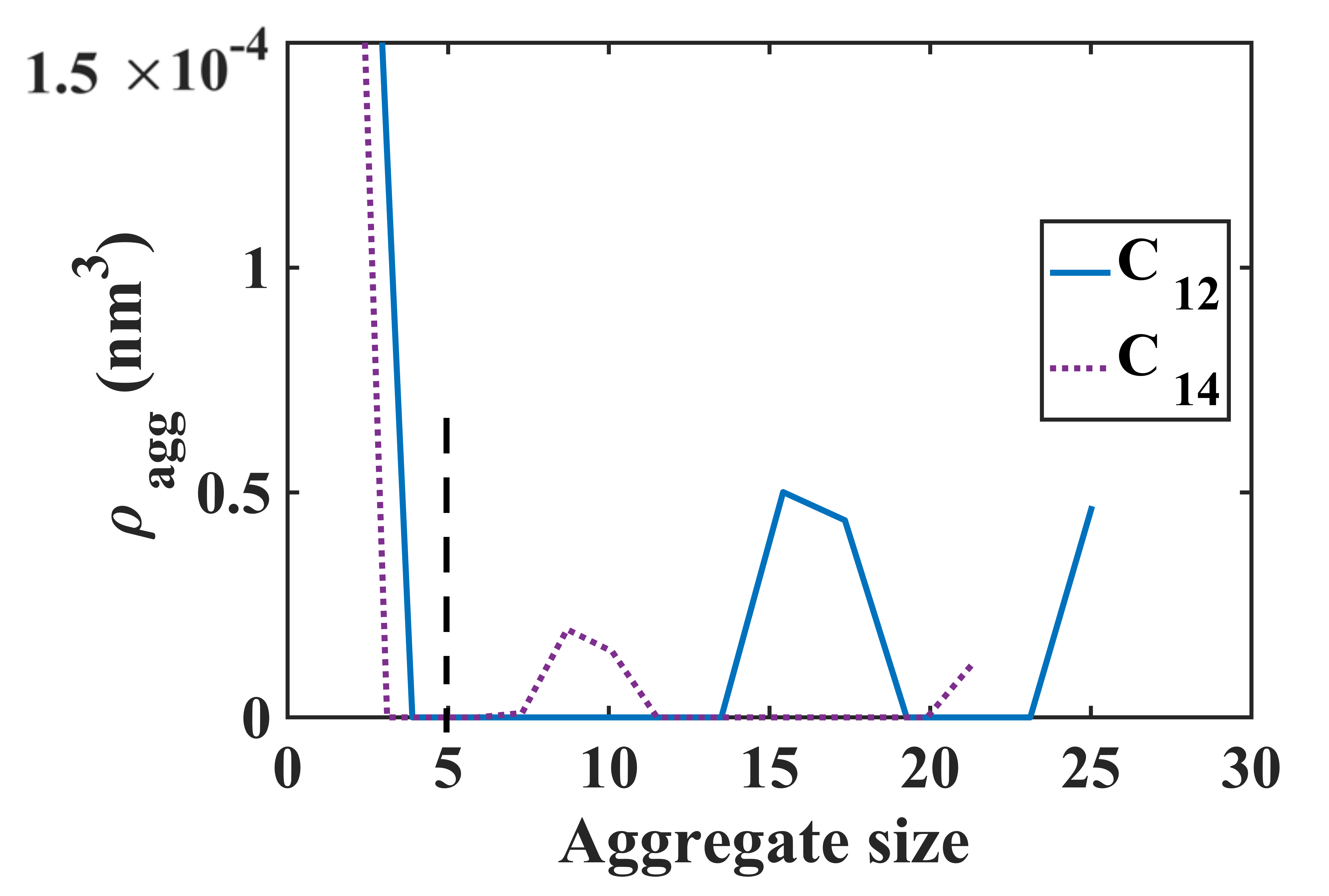

Supplement: Supplementary file 2 — High Resolution Image (TIF 805 kb) [file 12249_2018_1289_MOESM1_ESM.tif]

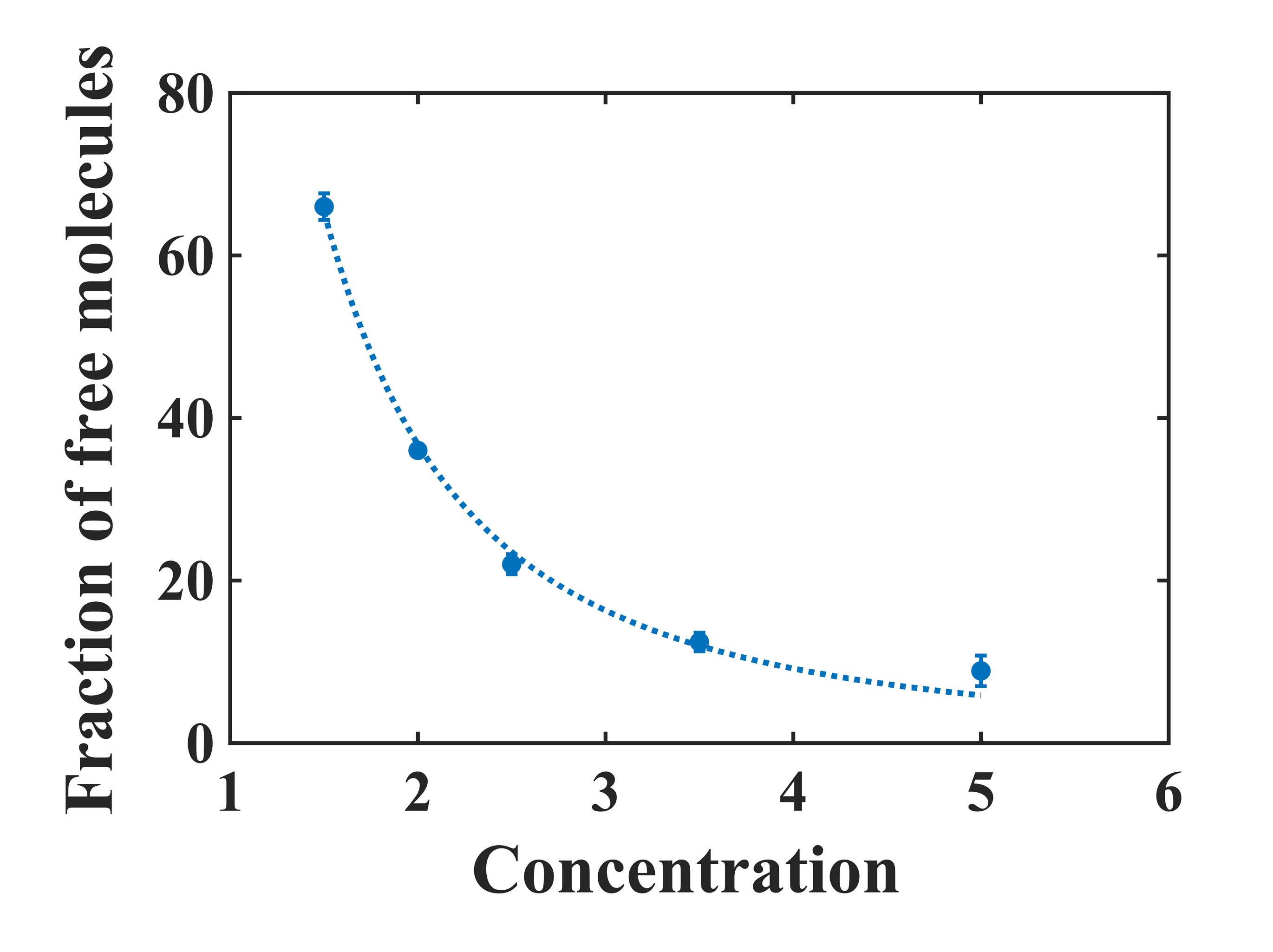

Supplement: Supplementary file 3 — Fraction of free molecules at different concentration level for C12 at 50C with 100% deprotonated molecules and 140 mM NaCl. The dotted line shows the fitting with the power-law equation. (PNG 190 kb) [file 12249_2018_1289_Fig7_ESM.png]
